# Isolation, Characterization, and Transduction of Endometrial Decidual Tissue Multipotent Mesenchymal Stromal/Stem Cells from Menstrual Blood

**DOI:** 10.1155/2013/901821

**Published:** 2013-03-31

**Authors:** Filippo Rossignoli, Anna Caselli, Giulia Grisendi, Serena Piccinno, Jorge S. Burns, Alba Murgia, Elena Veronesi, Pietro Loschi, Cristina Masini, Pierfranco Conte, Paolo Paolucci, Edwin M. Horwiz, Massimo Dominici

**Affiliations:** ^1^Division of Oncology, Department of Medical and Surgical Sciences of Children & Adults, University Hospital of Modena and Reggio Emilia, Via del Pozzo 71, 41100 Modena, Italy; ^2^Department of Internal Medicine and Oncology, University of Bari Aldo Moro, Bari, Italy; ^3^Unit of Plastic Surgery, Department of Medical and Surgical Sciences of Children & Adults, University Hospital of Modena and Reggio Emilia, Via del Pozzo 71, 41100 Modena, Italy; ^4^Division of Pediatric Oncology, Hematology and Marrow Transplantation, Department of Medical and Surgical Sciences of Children & Adults, University Hospital of Modena and Reggio Emilia, Via del Pozzo 71, 41100 Modena, Italy; ^5^Division of Oncology, Children's Hospital of Philadelphia, Philadelphia, PA 19104, USA

## Abstract

Mesenchymal stromal/stem cells (MSCs) reveal progenitor cells-like features including proliferation and differentiation capacities. One of the most historically recognized sources of MSC has been the bone marrow, while other sources recently include adipose tissue, teeth, bone, muscle, placenta, liver, pancreas, umbilical cord, and cord blood. Frequently, progenitor isolation requires traumatic procedures that are poorly feasible and associated with patient discomfort. In the attempt to identify a more approachable MSC source, we focused on endometrial decidual tissue (EDT) found within menstrual blood. Based also on recent literature findings, we hypothesized that EDT may contain heterogeneous populations including some having MSC-like features. Thus, we here sought to isolate EDT-MSC processing menstrual samples from multiple donors. Cytofluorimetric analyses revealed that resulting adherent cells were expressing mesenchymal surface markers, including CD56, CD73, CD90, CD105 and CD146, and pluripotency markers such as SSEA-4. Moreover, EDT-MSC showed a robust clonogenic potential and could be largely expanded *in vitro* as fibroblastoid elements. In addition, differentiation assays drove these cells towards osteogenic, adipogenic, and chondrogenic lineages. Finally, for the first time, we were able to gene modify these progenitors by a retroviral vector carrying the green fluorescent protein. From these data, we suggest that EDT-MSC could represent a new promising tool having potential within cell and gene therapy applications.

## 1. Introduction 

Mesenchymal stromal/stem cells (MSCs) are adult progenitor cells isolated from several human adult and perinatal tissues [1, 2]. While the acronym MSC seems to equate the biological properties of these useful progenitors, the possibility to isolate these cells from different tissue has been outlining common features combined with source-specific peculiarities that are still under investigation.

MSC demonstrated a positive impact in several pathological conditions [1, 3, 4], exerting their therapeutic functions on damaged tissues through different mechanisms, including differentiation into mature cells and largely obscure paracrine effects [5]. Moreover, MSCs have also been investigated for their possible use to deliver wild-type or gene modification-induced bioactive molecules with promising, but still undefined, influence in cancer models [6–9]. In the light of these findings, it appears reasonable to propose that a tissue source of MSC could be deemed relevant and useful by determining if it provides cells with varied differentiation potential and distinct cytokine profiles that may indicate an advantageous role in the interaction with tumors [1, 10]. Therefore, understanding these features from distinct MSC tissue sources shall be a primary objective to efficiently translate these cells into different clinical applications. 

One most recognized source of MSC has been the bone marrow obtained from iliac crest and more recently, MSC progenitors have been isolated from lipoaspirates and other tissue sources including teeth, bone, muscle, placenta, liver, pancreas, umbilical cord, and cord blood [11]. In the majority of these cases, especially the autologous sources, tissue isolation requires traumatic procedures sometimes linked with patient's discomfort.

In the attempt to identify a different and more approachable MSC source for cell and gene therapies, we focused on endometrial decidual tissue (EDT) obtained from menstrual blood. Previous cloning studies of isolated human endometrial cells provided early evidence of rare clonogenic mesenchymal cells, representing approximately 1% of endometrial cells suspension obtained by uterine tissue digestion after hysterectomy [12]. These endometrial stromal elements demonstrated properties similar to bone marrow and adipose tissue MSC including substantial self-renewal ability *in vitro*, high proliferative potential, and multilineage differentiation [13, 14]. However, obtaining these cells directly from endometrium either after hysterectomy or by biopsy still implies invasive procedures.

In order to evaluate the possibility of obtaining multi-potent cells from the uterus by a non-invasive and reproducible manner, researchers started to analyze shed menstrual blood and tissue in the attempt to identify menstrual blood-derived MSC [15, 16]. These pilot studies confirmed what was already known for endometrial cells obtained from hysterectomy, suggesting that endometrial stem/progenitor cells may be shed in menstrual blood. 

Following these early findings and by different isolation steps, this study characterizes EDT as a novel tissue source of MSC with regards to possible applications in regenerative medicine and gene therapy. MSCs from different sources have been investigated for their possible uses as tumor-specific delivery vehicles for suicide genes, oncolytic viruses, or secreted therapeutic proteins [8]. The mechanisms through which MSCs are considered to home to tumors are not completely clear, but seem to be dependent on biological properties of tumor microenvironment as well as the native tropism of selected MSC and also on the experimental procedures used [10, 17]. Thanks to genetic manipulation techniques, this natural tropism has been recently and convincingly exploited to transform MSC in “bullets” capable to deliver oncolytic viruses or various anticancer agents directly into tumor sites [8]. The possibility of having noninvasive procedures to obtain autologous progenitor cells, although with a gender limitation, paves the way to a more intense research activity aimed to deeply characterize MSC from a different source for selected biomedical applications. 

## 2. Materials and Methods 

### 2.1. Cell Procurement and Processing

Menstrual blood was collected from healthy female volunteer donors (*n* = 3) during the first few days of the cycle. Written consent was obtained from each donor and the Local Ethical Committee approved cells donation for research purposes. Each donor has been endowed with a menstrual cup (DivaCup, Diva International, San Francisco, CA, USA) to collect blood, which was transferred in phosphate buffered saline (PBS, PAA Laboratories, Pasching, Austria) with 1% penicillin/streptomycin (10,000 U/mL Penicillin, 10* *mg/mL Streptomycin in 0.9% NaCl solution, PAA Laboratories), 35* *mg/mL fluconazole (Diflucan, Pfizer, New York, NY, USA) and heparin (500 U/mL, Sigma, St. Louis, MO, USA). Samples were maintained at 4°C for 24–48* *h after procurement until reaching the processing laboratory.

The endometrial tissue, if present, was discarded, and the remaining blood was homogenized by 20 passages through a 19G needle using a 10 mL syringe. Cell suspension was then cultured as below reported. In addition, two alternative isolation protocols involving separation of the corpuscular fraction of blood through normal or density-gradient centrifugations were initially introduced. However, these latter two approaches were discontinued because of poor isolation efficiency (data not shown).

### 2.2. Cell Culture

Isolated cells were seeded into culture flasks to obtain an adherent fraction by adding *α*MEM (Gibco), 1% L-glutamine (200* *mM in 0.85% NaCl solution, Lonza Verviers, Belgium), 1% penicillin/streptomycin (PAA Laboratories), and 10% fetal bovine serum (FBS; PAA Laboratories). In addition, a serum-deprived medium (Quantum 333) with 1% penicillin/streptomycin was also introduced (all from PAA Laboratories). Cells were cultured for 7 days, washed with PBS (PAA Laboratories) to remove the nonadherent fraction, and fresh medium was added. At confluence, cells were detached by trypsin (trypsin 0.05% EDTA 0.02% in PBS, EuroClone, Milan, Italy) and subcultured at a density of 6000 cells/cm^2^ until functional assays. All flasks were incubated at 37°C, 5% CO_2_, and medium was changed every other day.

### 2.3. Clonogenic Assay

Adherent cells, out of passage 1 (P1) or passage 2 (P2), were seeded at clonal density of 100 cells/cm^2^ in *α*MEM (Gibco), 1% L-glutamine (Lonza), 1% penicillin/streptomycin (PAA Laboratories), and 10% FBS (PAA Laboratories). Colony formation was monitored daily. On day 10, cells were fixed with cooled absolute methanol for 2 minutes and stained for 5 minutes with 1% crystal violet aqueous solution (Sigma). Colonies with more than 50 cells were then counted, as originally described for marrow MSC [18]. Each assay was repeated in triplicate, and cloning efficiency (*E*) was calculated as *E*% = (n. clones/cells seeded) · 100.

### 2.4. Proliferation Assay

Adherent cells were seeded at a density of 6000 cells/cm^2^ in *α*MEM (Gibco), 1% L-glutamine (Lonza), 1% penicillin/streptomycin (PAA Laboratories), and 10% FBS (PAA Laboratories) in different flasks for the following time points: 24, 48, 72, and 96 hours. At each time point, cells were trypsinized and counted. Data obtained were plotted as number of harvested cells (*y*) against hours of culture (*x*), and the exponential growth curve was generated using GraphPad Prism software (GraphPad Software Inc., version 5.00). The doubling time (*T*) was obtained from the growth constant (*k*) of the exponential equation *y* = *a* · *e* ^*kx*^, where *k* = ln⁡⁡2/*T*.

### 2.5. Senescence-Associated *β*-Galactosidase Staining

To assess cell senescence, the activity of *β*-galactosidase (*β*-gal) at pH 6 was evaluated. *β*-gal expression is a feature of senescent cells [19]. According to Senescence *β*-galactosidase Staining Kit (Cell Signaling Technology, Beverly, MA, USA), growth medium was removed, and cells were washed with PBS (PAA Laboratories). Cells were then incubated for 15 minutes with a fixative solution (20% formaldehyde, 2% glutaraldehyde in 10x PBS) and washed twice with PBS (PAA Laboratories). Color development was obtained by incubation overnight at 37°C with the provided staining solution (40* *mM citric acid/sodium phosphate pH 6.0, 150* *mM NaCl, 2* *mM MgCl_2_, 5* *nM potassium ferricyanide, and 1* *mg/mL X-gal in DMSO). Plates were then observed by microscopy for the development of blue color.

### 2.6. FACS Analyses

Adherent cells were harvested for surface antigen analysis. Briefly, cells were detached from plastic support by trypsin (EuroClone), counted, and aliquoted in FACS analyses polypropylene tubes (0.5–1 · 10^6^ cells/tube) (VWR, Milan, Italy). EDT-MSCs were subsequently incubated in blocking buffer (100 *μ*L each 0.5–1 · 10^6^ cells) containing Dulbecco's Modified Eagle's Medium (DMEM, Gibco), 10% FBS (PAA Laboratories), and 0.1* *M sodium azide and human immunoglobulin G (both from Sigma) and incubated for 20′ on ice. After a PBS (PAA Laboratories) washing step, cells were resuspended in PBS (PAA Laboratories) with 0.5% bovine serum albumin (BSA, Sigma) and stained on ice and in the dark for 30′ with the following monoclonal antibodies: APC-anti-CD45, FITC-anti-HLADR, PE-anti-CD34, PE-anti-CD14, and FITC-anti-CD56 (all from Becton Dickinson, Franklin Lakes, NJ, USA); PE-anti-CD31 (BioLegend); APC-anti-CD90, PE-anti-SSEA-4 (both from eBioscience, San Diego, CA, USA); FITC-anti-CD105, PE-anti-CD73 (all from BD Pharmingen); APC-anti-CD146 (Miltenyi Biotec). In all the experiments, the corresponding isotype-matched antibodies were used as negative controls (BD Pharmingen and Becton Dickinson). Data were collected using a FACS Aria III flow cytometer (BD Biosciences) and analyzed on FACS Diva software (BD Biosciences).

### 2.7. Multilineage Differentiation Assays

To assess *in vitro* differentiation capacities, adherent cells after P2 were cultured in specific induction media. Media were changed every other day and undifferentiated controls were concurrently cultured in *α*MEM (Gibco), 1% L-glutamine (Lonza), 1% penicillin/streptomycin (PAA Laboratories), and 10% FBS (PAA Laboratories) for the same incubation time. Each assay was performed in triplicate.

Adipogenic, osteogenic, and chondrogenic differentiations were performed as previously reported [20]. Briefly, for osteogenic differentiation, cells were seeded at the density of 10000 cells/cm^2^ and maintained in the growth medium until confluence. Culture medium was then substituted with the induction one, composed by *α*MEM (Gibco) supplemented with 10 nM dexamethasone (Sigma), 10 mM *β*-glycerol phosphate (Sigma), 0.1 mM L-ascorbic acid-2-phosphate (Sigma), 10% defined FBS (Hyclone, Logan, UT, USA), 1% L-glutamine (Lonza), and 1% penicillin/streptomycin (PAA Laboratories). Induction was maintained for 14 days and, from the seventh day, 100 ng/mL BMP-2 (Peprotech, Rocky Hill, NJ, USA) was added. Confirmation of osteogenic differentiation was performed through Alizarin Red staining combined by real-time qPCR analysis (described below). At the end of the induction period, culture wells were washed briefly with a buffer solution containing 20 mM Tris-HCl and 150 mM NaCl in water. Cells were then fixed and dried with cooled absolute methanol for 2 minutes. After a washing step with ddH_2_O, cells were stained by 1.5% Alizarin Red aqueous solution pH 4.0–4.2 (Sigma) for 5 minutes and washed again first with ddH_2_O and then with PBS (PAA Laboratories) for 15 minutes. The last step consisted of dehydration using cooled absolute ethanol for 2 minutes followed by microscopic observation.

For adipogenic differentiation, EDT-MSCs were seeded at the density of 10000 cells/cm^2^ and maintained in the growth medium until confluence. The medium was then substituted with the induction one, composed of *α*MEM (Gibco) supplemented with 1 *μ*M dexamethasone (Sigma), 60 *μ*M indomethacin (Sigma), 10 *μ*M insulin (Sigma), 0.5 mM 3-isobutyl-1-methylxanthine (IBMX, Sigma), 10% rabbit serum (EuroClone), 5% horse serum (EuroClone), 1% L-glutamine (Lonza), and 1% penicillin/streptomycin (PAA Laboratories). Induction lasted 10 days and confirmation of differentiation was achieved through Oil Red O staining, as follows. Culture wells were washed briefly with PBS (PAA Laboratories). Cells were fixed with vapors of 37% formaldehyde (Sigma) for 10 minutes and then washed with water for 2 minutes. Staining was obtained adding an Oil Red O solution (10 mg/mL Oil Red O in ethanol 70% and acetone; all from Sigma) into the wells for 3 minutes. Excessive stain was removed by washing with water and cells were then counterstained by Harris hematoxylin (Bio-Optica, Milan, Italy) for 30 seconds.

Finally, for chondrogenic differentiation 2–5 · 10^5^ EDT-MSCs were aliquoted in a 2 mL tube and then centrifuged at 1200 rpm for 10 minutes. Cells were maintained pelleted in the growth medium at 37°C with the plug opened. After 2 days of incubation, tubes were centrifuged again, and the medium was substituted with the induction one, composed by *α*MEM (Gibco) supplemented with 100 nM dexamethasone (Sigma), 200 *μ*M L-ascorbic acid-2-phosphate (Sigma), 100 *μ*g/mL sodium pyruvate (Biochrom AG, Berlin, Germany), 40 *μ*g/mL proline (Sigma), 1x ITS+ premix (BD Biosciences, San Jose, CA, USA), 10 ng/mL TGF-*β* (Peprotech), 0.5 *μ*g/mL BMP-6 (Peprotech), 1% L-glutamine (Lonza), and 1% penicillin/streptomycin (PAA Laboratories). Before and after each medium change, tubes were centrifuged at 1200 rpm for 10 minutes. During the incubation period, cells remained as pellet with the tube plugs opened. Induction lasted 21 days, and specimens were fixed for 1 hour in 10% formaldehyde (Sigma) and then dehydrated by serial passages into ethanol at increasing concentrations, from 70% to 100%. Samples were then included into paraffin blocks and cut in slices on microscope slides for Alcian Blue staining. Slides were deparaffinized with the Histo-C cleaning agent (Celltech, Turin, Italy) and rehydrated through passages into a decreasing concentration alcoholic ladder (from 100% ethanol to 70% ethanol). Sample sections were then incubated with a 0.5 mg/mL Hyaluronidase (Sigma) in buffer phosphate solution (8 g/L NaCl, 2 g/L NaH_2_PO_4_, and 0.3 g/L Na_2_HPO_4_) with 10 mg/mL BSA (Sigma). Slides were washed in water for 5 minutes and then immersed in a 3% acetic acid solution for few seconds. Staining with 10 mg/mL Alcian Blue solution in 3% acetic acid (pH 2.5, Sigma) lasted 30 minutes, and after a washing step in water, samples were counterstained for 5 minutes with nuclear fast red solution (Sigma) and then washed in water.

### 2.8. Real-Time qPCR

Total cellular RNA was isolated from osteogenic committed and uncommitted cells, using Trizol reagent method (Invitrogen) according to manufacturer's instructions. Samples were concentrated by ethanol precipitation and suspended in RNase-free water. RNA quantity was assessed by spectrophotometry (DU730 UV/VIS Spectrophotometer; Beckman Coulter, Milan, Italy). A 2 *μ*g aliquot was reverse transcribed into cDNA using RevertAid First Strand cDNA Synthesis Kit (Fermentas) by oligo (dT)_18_ primers in a final volume of 20 *μ*L. Reactions were performed at 42°C for 1 h with a final step at 70°C for 5 minutes. cDNA was then used to determine the osteoblast-associated gene expression by quantitative real-time PCR technique using Step One Real-Time PCR System Thermal Cycling Block (Applied Biosystems, Foster City, CA, USA). Each sample was compared with noninduced control for the expression of alkaline phosphatase, collagen 1A2, and osteocalcin. *β*-actin was used as a reference gene. All primers were purchased from Integrated DNA Technologies, and sequences are reported in Table 1. PCR was performed with Fast SYBR Green Master Mix (Applied Biosystems) which uses AmpliTaq Fast DNA Polymerase, SYBR Green I dye to detect double-stranded DNA, and ROX dye as a passive internal reference. Reaction proceeded through an initial step at 95°C for 20′′, followed by 40 cycles of denaturation (3′′ at 95°C), annealing, and extending (30′′ at 60°C). The final stage comprises the analysis of the melt curve through a denaturing step (15′′ at 95°C) followed by annealing (1′ at 60°C) and ramping to 95°C with 0.3°C increment/step. Levels of mRNA for tested genes were quantified using ΔΔCT method and normalized against human *β*-actin as a housekeeping gene. Data have been analyzed by StepOne software (version 2.1, Life Technologies Corporation, Carlsbad, CA, USA). 

### 2.9. Gene Modification of EDT-MSC

Cultured cells were infected by a bicistronic murine stem cell virus-derived retroviral vector (pMIGR1) encoding for green fluorescent Protein (GFP). Retrovirus production was performed by the FLYRD packaging cell lines, as published by Marx et al. [21]. FLYRD cells were seeded in a T175 flask with 10* *mL of medium composed by DMEM (Gibco) supplemented with 10% defined FBS (Hyclone). Cell supernatant was collected and filtered (PES) with a 0.45 *μ*m filter, and EDT-MSC were incubated for 6* *hrs with 5* *mL culture medium, composed by: *α*MEM (Gibco), 1% L-glutamine (Lonza), 1% penicillin/streptomycin (PAA Laboratories), and 10% FBS (PAA Laboratories) with the addition of 5* *mL viral particles-containing supernatant and 6 *μ*g/mL polybrene (Sigma). Cells were then washed by PBS (PAA Laboratories) and culture medium was changed. The infection step was repeated for three consecutive days at which time cells were evaluated at FACS Aria III flow cytometer (BD Biosciences) for GFP protein expression. 7-amino-actinomycin D (7AAD) staining was also performed to evaluate mortality after transduction. Cells were evaluated by FACS Aria III (BD Biosciences), and data were analyzed using FACS Diva software (BD Biosciences).

### 2.10. Statistics

Data are expressed as average values, and analyses were performed by GraphPad Prism software. *t*-test was considered as significant with *P* value <0.05. 

## 3. Results 

### 3.1. EDT-Derived Cells Are *In Vitro* Heterogeneous but Retain Predominant MSC Features

**Figure 1:**
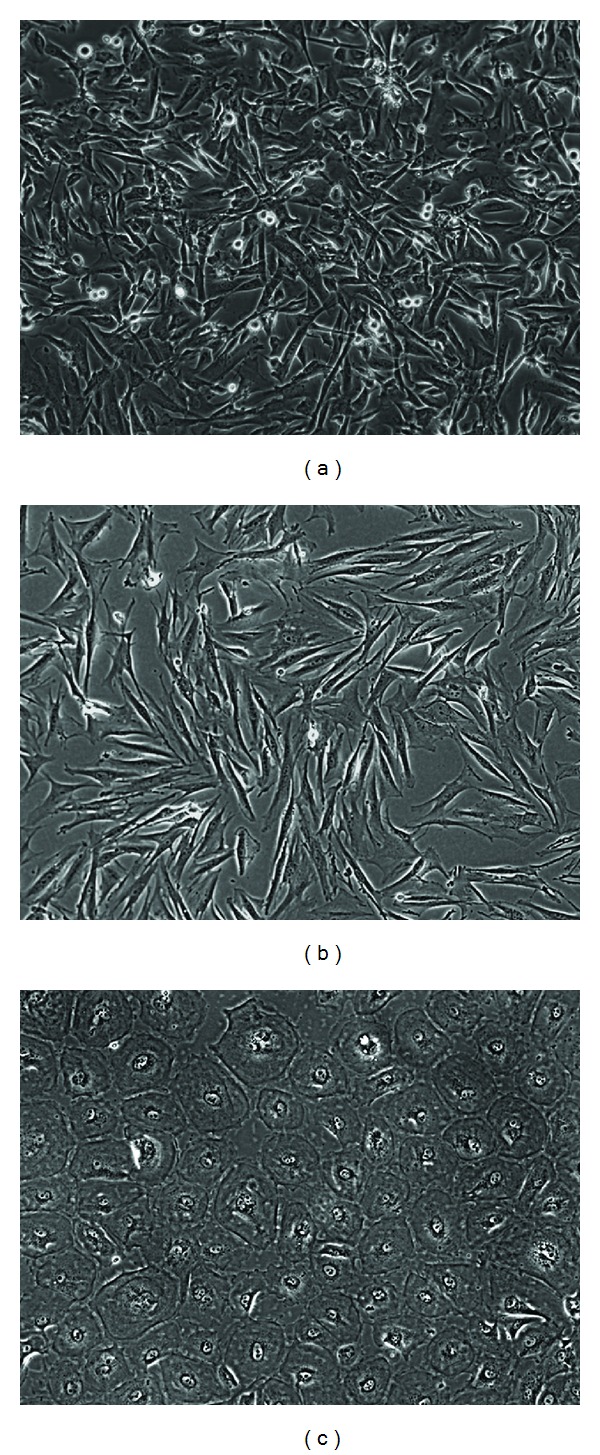
*In vitro* adherent cells from decidual tissues. (a) Representative photomicrograph of spindle-shaped adherent cells isolated by *α*MEM and 10% FBS. (b) Cells isolated *in vitro* by serum-deprived medium (Quantum 333) at early passages. (c) Another population of cells was *in vitro* isolated contextually with EDT-MSC. These elements share similarities with endothelial cells forming a monolayer of polygonal, sometimes binucleated cells. Original magnification 100x.

Adherent cells isolated from menstrual blood initially displayed *in vitro* fibroblast shape morphology in both *α*MEM with 10% FBS and Quantum 333 (Figures 1(a)-1(b)). However, prolonged cultures in *α*MEM with 10% FBS showed a significant better growth performance, and this medium was then preferentially used for all subsequent functional analyses. Adherent elements had typical mesenchymal aspect being fibroblastoid-like, spindle-shaped cells with an elongated cytoplasm. Within this population, it was also possible to identify more infrequent cell clusters with a distinct morphology. These appeared as endothelial-like sometimes binucleated cells, forming a monolayer with polygonal shape (Figure 1(c)). However, we did not confirm the nature of this population, because their presence disappeared after very early passages and because they were not the subjects of this study.

### 3.2. EDT-Derived Cells Show Robust Clonogenic and Proliferative Potential

**Figure 2:**
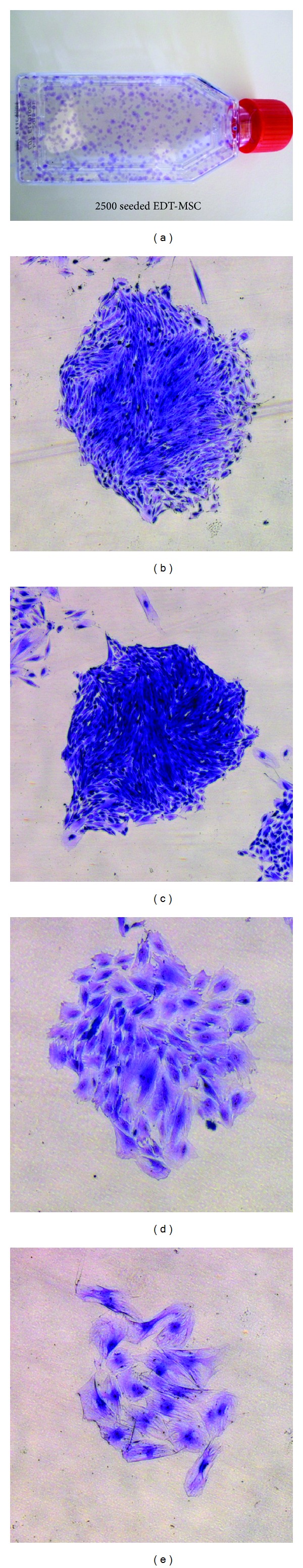
EDT-MSC clonogenic precursors. (a) A representative T-25 culture flask with crystal violet-stained EDT-MSC colonies. (b) and (c) EDT-MSC compact clones with small size cells. (d) and (e) EDT-MSCs could also generate less populated colonies with larger cellular elements. Original magnification 50x.

Having observed the fibroblast shape of EDT isolated cells, we then focused on their clonogenic and proliferative potential. We observed a high clonal efficiency with an average of 14.1% (10.8–17.9%) of the seeded cells able to generating colonies. This result indicates that the menstrual-derived cell population contains a large fraction of actively cycling cells with signficant clonogenic potential (Figure 2(a)). Of interests, colonies did not appear homogenous, and we were able to identify at least two kinds of morphologies. On the one hand, densely populated clones constituted by small size cells (Figures 2(b)-2(c)) and on the other, smaller cell clusters with elements having a large cytoplasm with an evident cytoskeleton (Figures 2(d)-2(e)).

**Figure 3:**
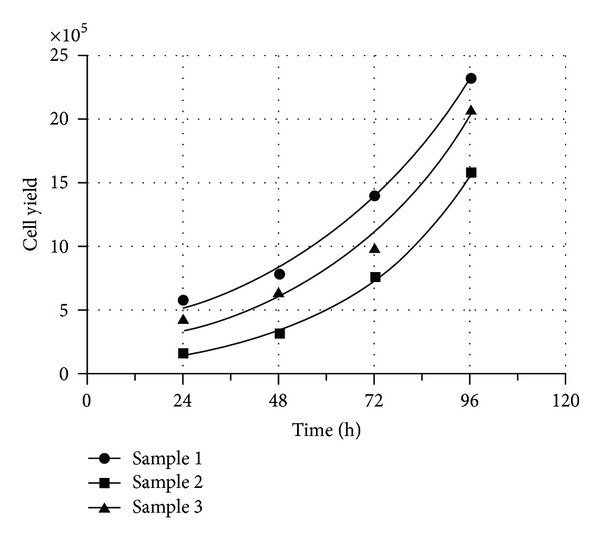
EDT-MSC proliferation. Cell proliferation assays performed to assess EDT-MSC doubling time. Doubling time for sample 1 was 33.0 h, for sample 2 was 21.9 h, and for sample 3 was 27.9 h.

**Figure 4:**
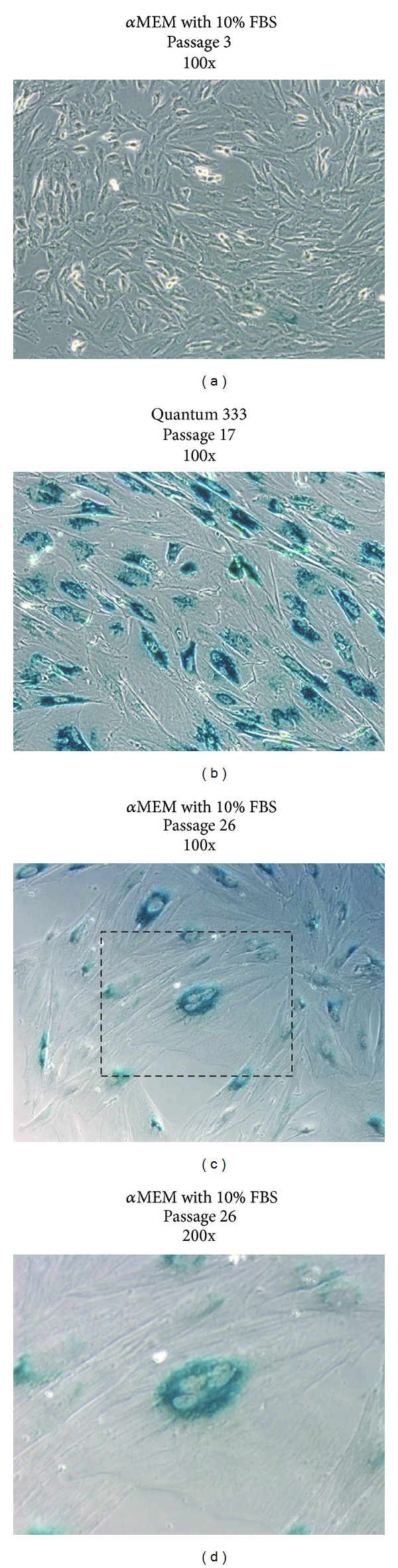
*β*-galactosidase staining. (a) Activity of *β*-galactosidase at pH 6 was assessed on passage 3 EDT-MSC cultured in FBS resulting almost negative for the presence of typical blue senescent cells. The conspicuous number of mitotic event is also visible, highlighting an intense proliferative activity. (b) Cells grown in Quantum 333 underwent senescence at passage 17 and resulted in deep blue staining within most of the amplified EDT-MSC. (c) Cells cultured in FBS were capable to reach passage 26 before showing senescence features, such as blue staining, large cytoplasm, multinucleated elements, and evident cytoskeleton. (d) A detail of a senescent cell. Original magnification 100x.

Isolated and expanded cells also demonstrated surprisingly low doubling time, with an average value of 27.6 hours (21.9–33.0) (Figure 3). The high number of passages that these cells were able to reach, further supported this remarkable growth property. The *β*-galactosidase staining, performed to evaluate senescent cells, showed that cells cultured in Quantum 333 underwent senescence at passage 17, while those cultured in *α*MEM supplemented with 10% FBS reached passage 26 before growth arrest (Figures 4(a)–4(d)). Together these data suggest how plastic-adherent, fibroblast shaped cells from EDT retain a strong proliferative potential comparable or even superior to MSC from other sources.

### 3.3. EDT-Derived Cells Display MSC Phenotypic Features

**Figure 5:**
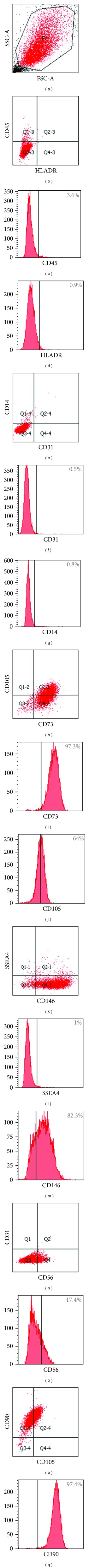
EDT-MSCs express typical MSC markers. Representative FACS analyses of EDT-MSC immunophenotype. Percentages are referring to the average positivity for each marker. Cells were gated out of 7-AAD positivity to exclude nonviable elements.

**Figure 6:**
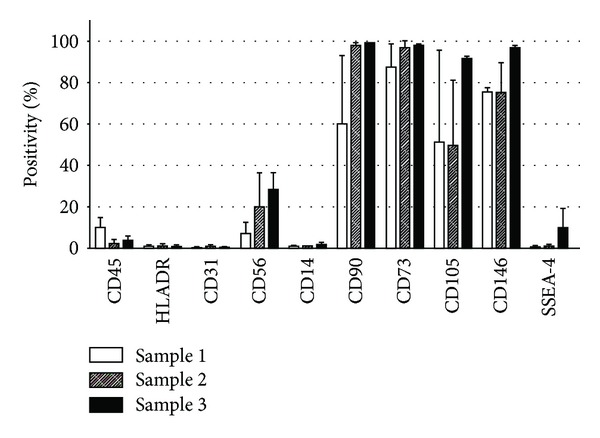
Overall EDT-MSC antigens expression after passage 1 *in vitro*. Marker expressions are here reported as average percentage (±standard deviation) of each tested sample.

Having evaluated the proliferation potential and to more carefully define the MSC nature of isolated and expanded cells, we assessed their antigen expression profile. EDT-derived cells express typical MSC markers (Figures 5 and 6), and in particular more than 90% of tested cells were positive for CD90 and CD73, constituting main features of MSC from other sources [22]. We also observed that more than 80% of cells expressed CD146, an adhesion molecule related but not restricted to MSC and also expressed by endothelial cells [1]. The levels of CD45, HLADR, CD31, and CD14, assessed early in culture and commonly used to distinguish MSC from hematopoietic and endothelial cells, were below 2% in most cases. In sample 1, we observed a slightly increase in the CD45^+^ fraction, suggesting the presence of hematopoietic elements which might have been isolated together with EDT-MSC. Other markers were also considered, such as CD56 whose positivity was extremely variable by up to 45%. Collectively, EDT-MSC from different donors showed the same overall trend in markers expression (Figure 6), despite for some markers, such as for CD56, CD105, and CD146, the variability has been considerably high. The phenotypic heterogeneity was also confirmed by analysis of physical parameters by FACS. Forward scatter versus side scatter plot was very dispersed, and it was impossible to identify consistent cell groups with similar physical parameters. Interestingly, we have to report a consistent small fraction (>1.0%) of SSEA-4 positive cells that reached 19.4% suggesting the expression of a pluripotency marker in this MSC type. 

### 3.4. EDT-MSCs Are Precursors of Three Mesenchymal Tissues *In Vitro*

**Figure 7:**
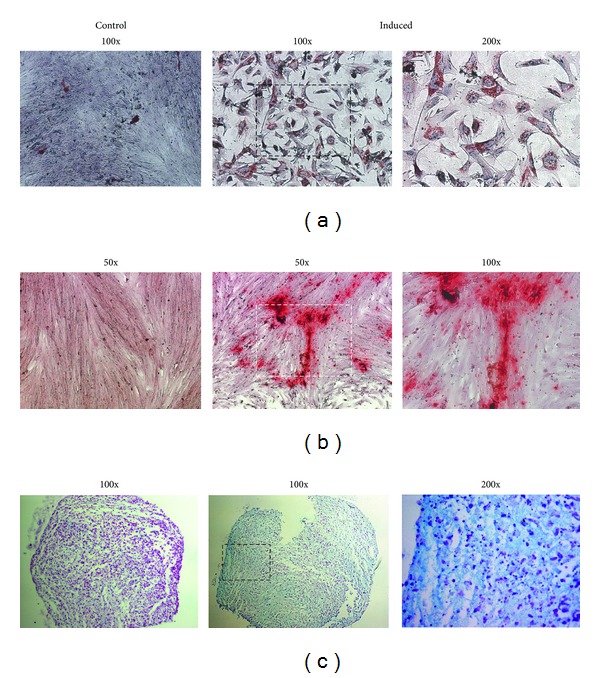
EDT-MSCs differentiate into committed mesenchymal tissues. (a) Oil Red O staining in adipogenic induced and control EDT-MSC. The red-brown vacuoles demonstrate differentiation into fat-producing cells. Original magnification 100x. (b) Alizarin Red staining showing calcium deposits in induced cells when compared with noninduced controls. Original magnification 50x. (c) Alcian Blue staining outlining sulfated proteoglycans expression suggesting differentiation into a chondrogenic tissue. Original magnification 100x.

**Figure 8:**
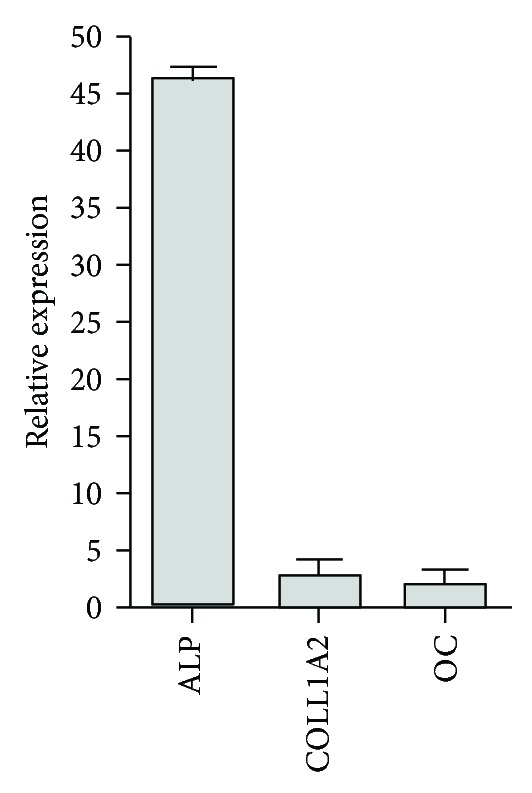
qRT-PCR for osteoblast-associated genes. The relative quantity of osteoblast-associated transcripts in induced EDT-MSC versus noninduced controls revealed an increased expression of the considered markers. All values were statistically significant (*P* < 0.05). ALP: alkaline phosphatase, COLL1A2: collagen 1A2, and OC: osteocalcin.

FACS analyses were then followed by assays aiming to assess EDT-MSC multipotency. We first focused on adipose commitment and, after 10 days of adipogenic inducing cocktail, EDT-MSCs were able to differentiate into vacuole-producing elements. The Oil Red staining confirmed the lipid content of those cells indicating the fat-producing ability of isolated and induced cells (Figure 7(a)). Osteogenic medium was then applied for 14 days, and cells underwent to osteoblastic commitment, forming a compact calcified matrix with calcium deposits confirmed by specific Alizarin Red staining (Figure 7(b)). Finally, after 21 days of chondrogenic induction, pellets of EDT-MSCs were included into paraffin blocks, and Alcian Blue highlighted the sulfated proteoglycans expression typical of the cartilaginous matrix, while Fast Red staining revealed the nuclei of resident chondrocytes derived from EDT-MSC (Figure 7(c)). Since a main focus of our research group is bone regeneration, we then coupled the cytochemical assay of osteogenic induced EDT-MSC with qPCR to further confirm bone commitment. As seen in Figure 8, all tested genes demonstrated an increased expression in induced samples versus noninduced controls. In particular, in osteoblast-induced cells the expression of alkaline phosphatase resulted 45 times higher (*P* < 0.0001), while more modest increases were observed for collagen 1A2 (2.9 times; *P* < 0.05) and osteocalcin (2.1 times; *P* < 0.05). These findings, together with positive Alizarin staining, reinforced the evidence for commitment of induced EDT-MSC to functional osteoblasts.

### 3.5. EDT-MSC Can Be Efficiently Gene Modified

**Figure 9:**
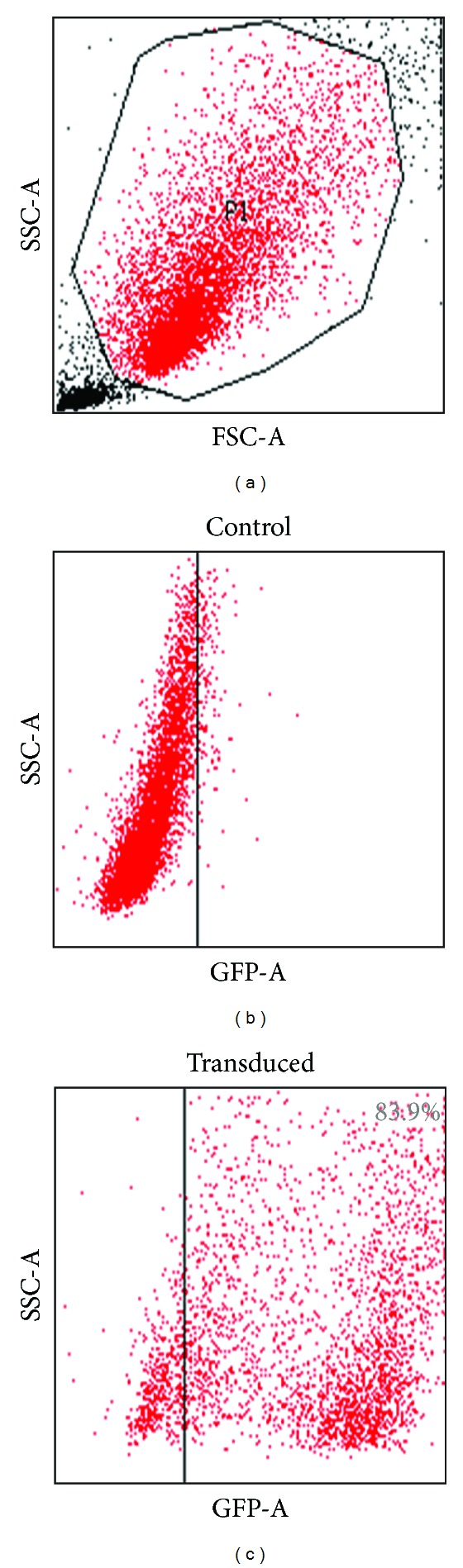
GFP expression of transduced and wild-type EDT-MSC. Representative FACS analyses assessing GFP positivity of wild-type control and transduced EDT-MSC.

To explore whether *ex vivo* expanded EDT-MSC could be genetically manipulated for future gene delivery approaches, cells were incubated with supernatants containing retroviral particles carrying the GFP gene. As shown in Figure 9, RD114 pseudotyped retroviral particles were able to efficiently and stably transduce EDT-MSC with levels greater than 80%, suggesting how this MSC type could be suitable for gene delivery approaches. 

## 4. Discussion 

Several MSC types have been obtained starting from different sources; here, we have isolated a population of mesenchymal progenitors from menstrual EDT and characterized them both at morphological and molecular levels investigating their ability to proliferate, to differentiate, and to be gene modified. EDT-MSCs appear as a heterogeneous cells population that can be safely and easily isolated by noninvasive manner, providing an expandable source of cells. In culture, they show mesenchymal morphology and, although some studies suggest they could derive from bone marrow [23], EDT-MSCs are generally considered to originate from the shedding of endometrial stem/progenitor cells which are mainly resident both in the basalis layer and, partially, in the functionalis area of endometrium [24].

The presence of a uterine population of progenitor cells would be consistent with the high tissue turn-over after each menstrual cycle. However, it is still uncertain how many subpopulations of progenitors are present in the endometrium and what are their specific properties. Therefore, in this pilot study, we began by addressing the existence of mesenchymal progenitors in the decidual endometrial tissue. 

The initial *in vitro* approach was performed to evaluate the clonogenic potential of these cells. EDT-MSC seeded at clonal density revealed the presence of two distinct colony types which closely resembled the stromal cell clones isolated by Chan et al. from endometrium after hysterectomy [12]. One was composed of smaller and densely packed cells, and the other was composed of larger and sparser elements. This finding seems peculiar for EDT-MSC and differs from colonies established from BM-MSC that appear larger and characterized by a central nucleus of cells surrounded by a crown of more sparse ones [18]. Next to qualitative evaluations of the colonies, our data suggest an average EDT-MSC clonogenic efficiency of 14%. This value is higher than what is described for BM-MSC and more similar or even superior to what reported for AT-MSC that retained around 10% of clonogenic precursors [25] (Table 3). In addition, our EDT-MSC population seemed to have a higher clonogenic efficiency compared to those described by other groups dealing with endometrial stromal cells whose values ranged from 1 to 11% [12, 24, 26]. While this may be due to donor-related issues such as age, *in vitro* technical aspects including harvesting and preservation procedures that may also drive these differences. Dimitrov et al. observed a tendency for these cells to decrease in clonal efficiency with the increase of seeding density [26]. Similarly, other culture conditions may generate better performing EDT-MSC. In this pilot study, we tested two different growth media, such as *α*MEM with 10% FBS and a serum deprived medium. Although these cells apparently did not shown particular nutritional requirements, serum-containing medium proved to be the best choice, ensuring a better morphological appearance and an improved longevity. Previous studies reported the use of different media such as DMEM/F-12 and Chang complete media often supplemented with calf serum, hormones, and growth factors [12, 14, 26, 27]. This wide range of culture conditions is likely to impact the *in vitro* behavior, leading to different performance in *ex vivo* pivotal parameters.

Using a FBS-based medium, EDT-MSC demonstrated a remarkable proliferation rate rapidly doubling their number in less than 28 hrs. This result agrees with other similar studies, which reported a doubling time between 18 and 36 h for EDT-MSC [15, 28]. As seen in Table 3, similar attitude has been also reported for dental pulp stem cells (DPSCs) and umbilical cord (UC) MSC that duplicate in approximately 30 hrs, considerably faster versus both BM-MSC and AT-MSC [29–31]. 

Beside this robust proliferative attitude, we also observed that plastic adherent fraction from shedded DT could be expanded for more than 25 passages before reaching senescence, a result once again higher than what reported for MSC from other sources. As summarized in Table 3, BM-MSC and AT-MSC senescence has been reported from passages 7 and 8 [32], while UC-MSC grow longer in culture, until passage 16 [31]. These findings reinforce the hypothesis that EDT-MSC can duplicate for long time before senesce and are consistent with reports asserting 25–30 population doublings for uterine-derived precursors [15, 28, 33]. This unique feature fit well with the high rate of renewal retained *in vivo* by the endometrium, leading to the conclusion that this new MSC source could have a tremendous potential for large-scale clinical applications in regenerative medicine.

In this work, EDT-MSC showed other similarities with MSC from the other sources and, in particular, under the immunophenotypical point of view satisfying recognized phenotypic criteria for MSC [22]. The biomarker profile, as compared in Table 2, shows a close resemblance of EDT-MSC with other sources MSC, expressing high levels of CD73 and CD90 and being negative for CD14, CD45, CD31, and HLA-DR. Other markers have been additionally evaluated. CD56, also known as neural cell adhesion molecule (NCAM), is a glycoprotein expressed by neuroectodermal-derived cells such as neurons and glia but also by skeletal muscle, NK cells and activated T cells [34]. Among our EDT-MSC, CD56 expression has been extremely variable (1.3–45%), and this may support the observation made by others exploiting CD56 expression to discriminate distinct marrow MSC subpopulations [35, 36]. Moreover, we evaluated the expression of SSEA-4, a glycolipid recently proposed to be a novel marker for BM-MSC isolation and that is expressed by teratocarcinoma cells and embryonic stem cells [37]. EDT-MSC demonstrated a noticeable expression of SSEA-4 that, while reaching 19.4% appeared far from the 90% positivity reported by others on decidual-derived cells [15]. However, different laboratories reported discrepancies on EDT-MSC phenotype [15, 28, 38], and this could be likely due to the heterogeneity of menstrual blood cell population, to differences in cell selection and, as stated, to culture conditions [13]. In addition, the relatively short time from the early evidence of these progenitors does not help in the phenotypical standardization and certainly will require deeper and multicentric studies.

Multipotency is another key feature of MSCs and all EDT-MSC lines showed the *in vitro* capacity to differentiate into three mesodermal lineages, such as osteoblast able to generate mineralized matrix, lipid vacuole-containing adipocyte and matrix-producing chondrocytes. Additional experiments will be necessary to confirm this ability, prompting their broad introduction in regenerative medicine applications. However, we have here begun to assess the capacity of these cells for robust osteogenic commitment confirmed by the differentiation data obtained by staining for mineralized matrix, we observed a relevant increase in the expression of typical osteoblast-associated genes such as alkaline phosphatase, osteocalcin, and collagen 1A2. 

Regenerative therapies based on stem cells, and more specifically on MSC infusion, may counteract tissue-losing states and may also be useful to substitute the congenital abnormal stem cell compartment in order to regenerate healthy functional cells. In this perspective, ease of supply, wide window of harvesting, and availability of EDT-MSC would allow autologous or, ultimately, allogeneic EDT-MSC transplantation. These properties have already been exploited, and EDT-MSC have demonstrated a therapeutic effect in a murine model of muscular dystrophy [38] and a neuroprotective effect in an experimental stroke model [27]. Moreover, endometrial-derived cells seem to have robust anti-inflammatory and immunomodulatory properties similarly to other MSCs [27, 39]. For this reason they were intravenously and intrathecally transplanted in 4 patients with multiple sclerosis, notably without arising immunological reactions or other adverse effects and with a clinical benefit that shall be carefully assessed [40]. 

Another original result we here report is the high transduction efficiency of EDT-MSC by a GFP-carrying retroviral vector. The procedure generated exogenous gene expression in more than 80% of the cells and, to our knowledge, this is one of the first reports of such highly efficient genetic manipulation of EDT-MSC by a single GFP-carrying vector providing a basis for use of these cells in gene therapy approaches, including targeted cancer treatment or their use as cell vectors for peptides or trophic factors. Having extensively characterized EDT-MSC, we presume that these cells may be useful for a more efficient delivery of proapoptotic molecules thanks to the high transduction efficiency and the rapid proliferation. This may allow a more targeted approach and better persistence of the cells in the tumor microenvironment. 

Starting from these findings, future studies shall focus on the characterisation of the heterogeneous cell populations identifiable in menstrual decidual tissue, investigating the existence of different progenitor subtypes as well as understanding the origin of these cells. More reliable techniques to isolate and cultivate pure populations of EDT-MSC should be developed and standardized in parallel to allow a rapid introduction of this promising therapeutic tool into the clinic. 

## 5. Conclusions 

This work highlights crucial features of a novel and still not completely understood population of mesenchymal progenitors isolated form endometrial decidual tissue. Cells grown *in vitro* were characterized by a rapid proliferation, a high clonogenic potential and a long-term survival. Advantages in comparison with MSC from other sources include a greater ease of supply and the protracted availability during a woman's lifetime with the additional benefit of deriving stem cells from a waste tissue, thus avoiding critical ethical issues. In the light of their capacity to differentiate into mesenchymal tissues and their propensity to undergo genetic manipulation, EDT-MSCs hold promise for novel therapeutic approaches for still incurable and highly disabling diseases.

## Figures and Tables

**Table 1:** Primer sequences.

Gene	Primer sequence	Amplified length
*β*-actin	5′-ACC TTC TAC AAT GAG CTG CG-3′ (sense)	148 bp
	5′-CCT GGA TAG CAA CGT ACA TGG-3′ (antisense)	
ALP	5′-GAT GTG GAG TAT GAG AGT GAC G-3′ (sense)	142 bp
	5′-GGT CAA GGG TCA GGA GTT C-3′ (antisense)	
COL1A2	5′-AGG ACA AGA AAC ACG TCT GG-3′ (sense)	146 bp
	5′-GGT GAT GTT CTG AGA GGC ATA G-3′ (antisense)	
OC	5′-CAG CGA GGT AGT GAA GAG AC-3′ (sense)	144 bp
	5′-TGA AAG CCG ATG TGG TCA G-3′ (antisense)	

ALP: alkaline phosphatase, COL1A2: collagen 1A2, OC: osteocalcin.

**Table 2:** Growth characteristics of MSC from different sources.

Cell type	Source	Isolation yield	Doubling time	Clonogenic efficiency	Senescence passage
BM-MSC	Bone marrow aspirate	60–600 cells from 1 mL of blood [41]	61.2 h [29]	3.9%*	7 [32]
PB-MSC	Peripheral blood	1 to 13 MSC from 1 million of mononuclear cells [11]	N/A	N/A	N/A
DPSC	Deciduous and adult teeth	N/A	30 h [30]	N/A	N/A
UC-MSC	Umbilical cord of newborns	5 · 10^4^–5 · 10^5^ cells from 1 cm^3^ of umbilical cord tissue [42]	24 h [31]	N/A	16 [31]
AT-MSC	Liposuction, lipectomy, and lipoplasty	5 · 10^3^ cells from 1 g of tissue [11]	45.2 h [29]	10% [25]	8 [32]
EDT-MSC	Decidual tissue	Variable^‡^	27.6 h^‡^	14.4%^‡^	26^‡^

Comparison between growth parameters of MSC from different sources. BM-MSC: bone marrow MSC; PB-MSC: peripheral blood MSC; DPSC: dental pulp stromal cells; UC-MSC: umbilical cord MSC; AT-MSC: adipose tissue MSC; EDT-MSC: decidual tissue MSC.*Unpublished data; ^‡^original data from this study, references.

**Table 3:** Immunophenotype of MSC from different sources.

Marker	CD14	CD45	CD73	CD90	CD105	CD146	CD31	HLA-DR	References
BM-MSC	−	−	+++	+++	+++	++	−	−	[43, 44]
PB-MSC	−	−	N/A	+++	++	N/A	−	−	[45]
DPSC	−	−	+++	+++	+++	N/A	−	−	[30]
UC-MSC	−	−	+++	+++	+++	N/A	−	−	[2, 43]
AT-MSC	−	−	+++	+++	++	N/A	−	−	[2, 43]
EDT-MSC	−	−	+++	+++	++	+++	−	−	‡

Comparison between positive and negative MSC surface markers from different sources, including EDT-MSC. Variable expression is highlighted with ±. BM-MSC: bone marrow MSC; PB-MSC: peripheral blood MSC; DPSC: dental pulp stromal cells; UC-MSC: umbilical cord MSC; AT-MSC: adipose tissue MSC; EDT-MSC: decidual tissue MSC. ^‡^Original data from this study, references.
